# A model for public–private partnership during the COVID‐19 pandemic: Lessons from Biolab and public laboratories working in the Hashemite Kingdom of Jordan

**DOI:** 10.1111/irv.13209

**Published:** 2023-10-18

**Authors:** Issa Abu‐Dayyeh, Zein Naber, Luke W. Meredith, Lora Alsawalha, Dana Nassar, Lara Sumrain, Mohammad Ghunaim, Thaer Hasan, Amid Abdelnour

**Affiliations:** ^1^ Department of Research and Development Biolab Diagnostic Laboratories Amman Jordan; ^2^ Infectious Hazard Management, Department of Health Emergency World Health Organization, Eastern Mediterranean Regional Office Cairo Egypt; ^3^ Jordan Country Office World Health Organization Amman Jordan

**Keywords:** Biolab, COVID‐19, Jordan, NGS, PCR, public–private partnerships

## Abstract

**Introduction:**

The global COVID‐19 pandemic overwhelmed national public health and laboratory capacity in Jordan and globally. In response, Biolab, a private laboratory group with 27 branches across Jordan, assisted with testing. Biolab was equipped to quickly increase molecular testing capacity without compromising quality or turnaround time, allowing them to contribute to national COVID‐19 surveillance efforts.

**Methods:**

Biolab expanded testing in Jordan by operationalizing automated testing platforms at various locations, including 16 branches, 2 drive‐through and 2 walk‐through centres, and entry points for airports and marine passenger arrivals. Genomic and molecular testing were implemented to track variants. Information technology platforms were introduced for sample management, registration, and commercial sample payments. Data were directly provided to the Ministry of Health through these platforms to support public health decision‐making and responses. Biolab prioritized staff well‐being by providing mental, financial, and physical health support during the pandemic.

**Results:**

Biolab processed more than two million samples, with a turnaround time of ~1.5 h. Results were transmitted directly to key stakeholders in near real time. Biolab conducted variant evaluations on >1.4 million samples using molecular variant testing and >1000 samples using whole genome sequencing. Biolab prioritized staff well‐being, improving staff satisfaction from 74% to 91%, a remarkable achievement when many laboratory systems experienced staff burnout and dissatisfaction.

**Conclusion:**

The collaboration between public and private laboratories during COVID‐19 established a model for future joint efforts to prevent outbreaks from becoming pandemics. Biolab's focus on efficiency, quality, and staff well‐being enabled consistent, high‐quality performance. The introduction of innovative information technology platforms ensured swift information dissemination. Biolab plans to continue investing in these platforms and expand pathogen testing, creating a top‐tier testing infrastructure in Jordan with a demonstrated ability to cooperate with the government for public benefit.

## 1 INTRODUCTION

In January 2020, the World Health Organization (WHO) declared the coronavirus disease (COVID‐19) as a public health emergency of international concern as the pandemic swept the world.^1^ To date, 763.74 million cases^1^ have been confirmed by testing through either rapid or molecular diagnostics, and 6.91 million people have died from COVID‐19‐related illness (Case Fatality Rate 0.9%), making it one of the worst pandemics in history.

The causative agent, severe acute respiratory syndrome coronavirus type 2 (SARS‐CoV‐2), is a coronavirus from the Sarbecovirus family, related to SARS‐CoV‐1 and Middle East Respiratory Syndrome.^2^ The virus emerged in China in 2019 but spread rapidly across the world, forcing public health institutions to respond with unprecedented steps such as lockdown and quarantine rules, testing mandates, and national and international vaccination programmes. Mandatory testing for symptomatic and asymptomatic individuals was a commonly used tactic for tracking the virus, resulting in severe stress on public health laboratories to meet the demand.

The Hashemite Kingdom of Jordan has a relatively small population of 11.2 million people and a small land area resulting in a population density of 300 people per square mile, comparable with France, one of the worst affected countries during the pandemic.^2^ During the pandemic, Jordan recorded 1.75 million cases, with 14,122 deaths per million people and 154,795 cases per million people.^1^ This made COVID‐19 the highest cause of death due to communicable disease in Jordan between 2020 and 2022. In response to the outbreak, a range of stringent nonpharmaceutical interventions was imposed by the national government. This included widespread lockdown and quarantine measures, as well as closing the borders to travellers,^3^ while providing easy access to health facilities, vaccination programmes, and widespread, high‐quality laboratory testing.

Collaboration between the private and public sectors, including commercial laboratory partners and academic institutions with laboratory capacity, was a cornerstone of the response in countries such as the United Kingdom and United States. These were often viewed as challenging, with the range of techniques, platforms, and assays causing difficulties in data standardization, while recruiting sufficient competent staff to maintain the necessary testing capacity was also a challenge many countries encountered.^1^ Jordan has a strong and established laboratory programme managed through the Ministry of Health (MoH) Public Health Laboratory Network. As in most countries, this was placed under extreme pressure due to the volume of tests required to keep up with the demand for symptomatic and asymptomatic cases across the country. To support the caseload, the MoH called upon private laboratories to contribute to diagnostic testing across the country as part of the national response to the pandemic.

Biolab was one of the first private laboratories called upon to support the testing effort. It is a chain of nationally and internationally accredited medical diagnostic laboratories established in 2001 to provide a full range of high‐quality testing services to Jordan. It has 27 branches, with 22 in Amman, 1 in Aqaba, 1 in Balqa, 1 in Zarqa, and 2 in Irbid. This made it an ideal partner for the MoH, providing geographically diverse laboratories with a wide range of capacities that could be rapidly integrated with the existing national laboratory response. Biolab also has an established and prolonged track record of performance, with over 22 years in the country, which means there are already links between the private laboratories and reporting mechanisms needed for outbreak response in the country. As soon as the pandemic was declared, Biolab initiated a crisis management policy and established a crisis management team with the primary objective of ensuring business continuity while scaling up operations to meet the anticipated pandemic demands. Key considerations included logistics and assuring supply chains were intact, the safety and security of staff, and updating and integrating IT infrastructure to ensure a seamless delivery of results to national databases in the MoH, as well as immediately back to patients, primary and tertiary healthcare facilities. Herein, we provide an overview of the steps taken and the key lessons and successes from the operationalization of COVID‐19 testing in Biolab over the course of the pandemic.

## 2 METHODS

### 2.1 Establishment of a diverse range of testing centres

**FIGURE 1:**
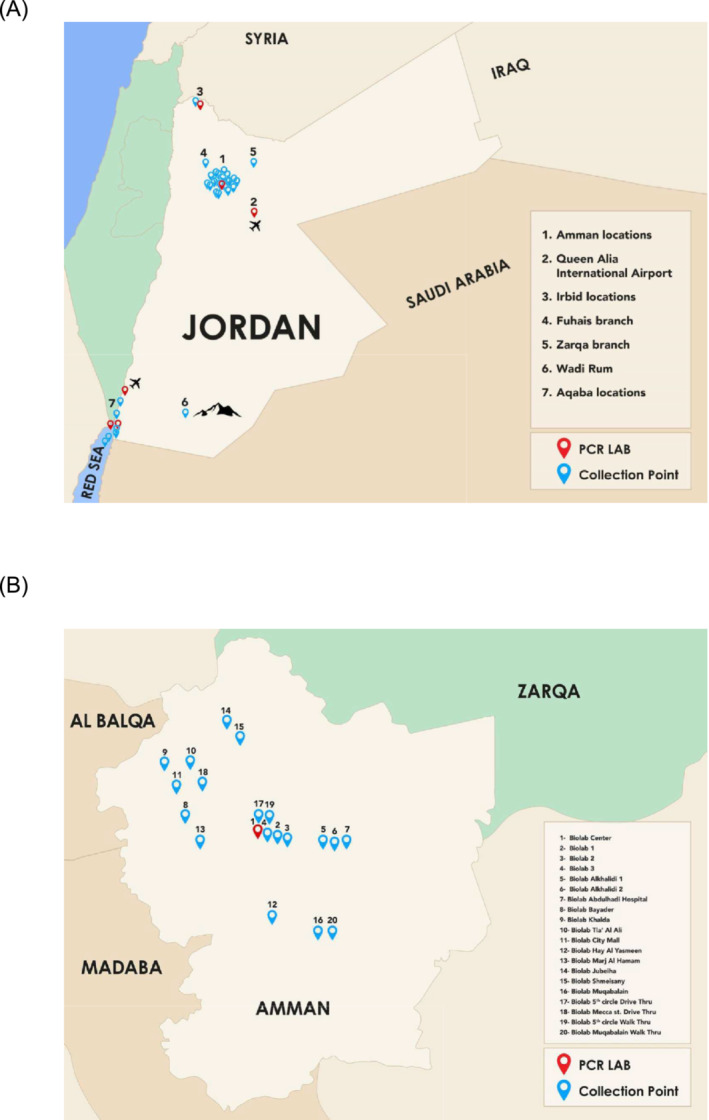
Biolab provided a geographically diverse laboratory network that tested over two million samples over the course of the pandemic. (A) Biolab established facilities in 36 locations across Jordan. This included both collection points and PCR laboratories for processing samples, in locations including border crossings, national entry points (airports and docks), and regional locations to ensure testing was accessible across the country. (B) In particular, 20 sites were located within Amman, the most densely populated city in Jordan.

MoH assigned Biolab to test all travellers and passengers arriving in Amman or Aqaba governorate, two of the busiest entry points to the country. To meet this requirement, 30 collection centres and six adjacent Polymerase Chain Reaction (PCR) laboratories were established in various parts of the country where our branches can be found and at all key entry points designated by the government and at selected remote areas (Figure 1). This included the Queen Alia International Airport, King Hussein Aqaba Airport, Aqaba Passenger Port Terminal to Egypt, Tala Bay port Terminal to Taba (Egypt), the Jordan/KSA border crossing at Durra, Wadi Araba border crossing, and Wadi Rum. Testing centres were also set up on tourist cruise ships operated by the Mediterranean Shipping Company, coming to the port of Aqaba. These cruises often sailed in the Red Sea for more than 3 days and therefore to abide by MoH regulations that a COVID‐19 PCR for anyone entering the country should be done within 24–48 h of arrival, passengers had to be tested before disembarking the ship to enter Jordanian soil.

### 2.2 COVID‐19 molecular diagnostics

To support high‐throughput testing, Biolab implemented an automated extraction and quantitative PCR workflow. Rapid extraction was carried out using the Zybio EXM3000 system, installed in the Molecular Departments at Biolab Center (Amman), Biolab Irbid branch, PCR laboratories at Queen Alia International Airport (Amman) and the King Hussein Airport (Aqaba), Aqaba Passenger Port Terminal to Egypt, and on deck of ships to support on‐site extraction to streamline the turnaround time. PCR assays were assessed, and ThermoFisher TaqPath COVID‐19 assay^4^ was selected as it met the quality and throughput requirements for the widely distributed testing network.

### 2.3 Variant assessment and analysis

Samples were tested for variants using customized variant‐specific TaqMan PCR assays. Variants of concern or interest assigned by WHO and Centres for Disease Control and Prevention were analysed and reported through the variants portal in Jordan and directly provided through daily, weekly, and monthly reports to the MoH to guide decisions regarding national public health interventions, including implementation of quarantine measures, allocation of medical personnel and resources, vaccination rollouts and campaigns, use of specific diagnostic assays, and treatment decisions for patients suffering acute COVID‐19 compared with other respiratory diagnoses.

### 2.4 Genomic surveillance

Biolab collaborated with Dr. Kristian Andersen at the SCRIPPS Institute, California, USA, and, later in 2021, with the Centers for Research in Emerging Infectious Diseases (CREID) West African Research Network for Infectious Diseases (WARN‐ID), to create a genomics‐based public health pathogen surveillance and outbreak response system for Jordan. The system utilized existing infrastructure, including Illumina MiSeq and Thermo Scientific Ion Torrent S5 platforms, and provided national staff with the necessary training and expertise to carry out genomics workflows. The SARS‐CoV‐2 virus was sequenced using PrimalSeq‐Nextera XT, adapted for Illumina Nextera XT library preparation or the Ion Torrent platform. Biolab staff underwent extensive training with experts from the Andersen laboratory. The resulting data were analysed locally and shared with relevant public health authorities nationally and globally through the Global Initiative on Sharing All Influenza Data (GISAID) framework for sharing of respiratory viral gene sequences/metadata. The data in GISAID are incorporated into national and international surveillance reports by multiple health agencies including the WHO.

### 2.5 Information technology

**FIGURE 2:**
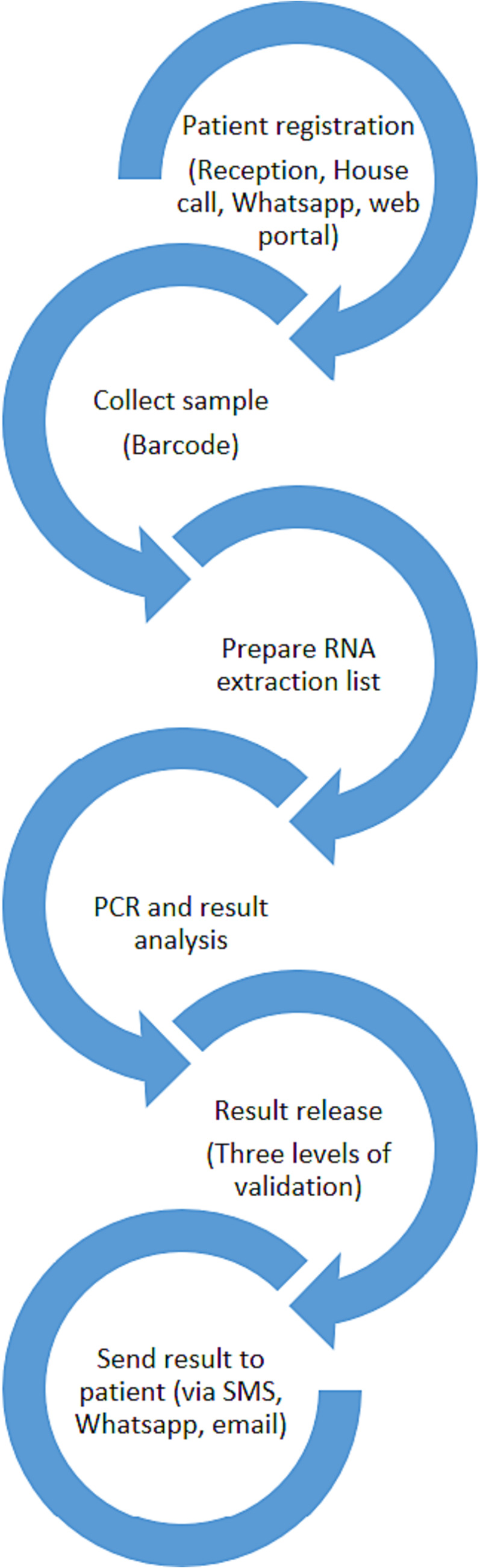
An integrated, end‐to‐end digital surveillance system was implemented to support the tracking of samples from collection to reporting. The COVID‐19 sampling and tracking platform implemented by Biolab's IT department had several key features, including sample tracking through the testing and reporting system using a unique QR code for each sample. Patients received this code at testing and then followed the sample through transport and reception at the laboratory. The system integrated with passenger entry/exit testing, enabling patients to register and track samples through the network using mobile applications. The platform ensured quality assurance by ensuring all quality metrics were within defined thresholds, validating results before release, and compiling and distributing reports. It also supported financial aspects of testing, including invoicing and internal cost allocations. Overall, the platform allowed for unprecedented visibility and tracking of sample testing and reporting.

The networked information technology (IT) solution implemented by Biolab provided a seamless and efficient means of tracking and distributing results across all locations (Figure 2). The end‐to‐end tracking of samples, from registration to reporting, allowed for rapid dissemination of results to patients, healthcare facilities, and public health authorities. The IT platform included standalone and mobile applications, making it easily accessible for Biolab staff, customers, and reporting agencies. The platform also allowed for automated release of results following the completion of quality controls, ensuring accurate and timely reporting of results. Three result validation levels were employed: the first by the technician performing the test, the second by the supervisor, and the third by the laboratory director, all of whom were authorized by the MoH through holding a valid professional practice license. Integrating the platform with reporting agencies such as the MoH and employers such as airlines or E‐visa systems further streamlined the process and facilitated efficient communication between relevant parties.

### 2.6 Staff healthcare and well‐being

Biolab's human resource (HR) department recognized the potential challenges that staff may face during the pandemic and implemented measures to support their physical, financial, and mental well‐being. In addition to financial incentives in the form of a 40% pay increase, staff who had close contact with samples or patients received further financial support through the COVID risk allowance. Biolab's HR department provided mental health support and increased paid vacation allowances to support staff in managing stress and maintaining their overall well‐being. Maternity leave was increased from 12 to 18 months, while nursery allowances were extended to the first 2 years following birth to ensure that mothers were well supported as well through the pandemic. Staff were encouraged to report contacts or infection and were provided with 24/7 support through their illness, including the establishment of on‐site staff mini‐clinics, provisioned with oxygen supplies, and medical support to ensure staff could access medical care while the hospitals were burdened with national cases.

## 3 RESULTS

### 3.1 Biolab provided rapid laboratory testing support across Jordan, with consistent quality and turnaround times

Over the course of the pandemic, through the implementation of the automated platform and expansion of testing across the country, the laboratory network tested 2,052,760 samples, with 985,069 originating from primary healthcare centres and 1,067,691 from entry/exit testing of passengers entering the country, providing support to both the national health system and primary patients (Figure 1). The average turnaround time was 1.5 h from the time it reached the laboratory. Of these samples, a high proportion came from drive‐through or public testing facilities.

### 3.2 Biolab provided high‐quality results, as demonstrated through excellent external quality assurance results

A common concern with private laboratory networks is the presence of “cowboy” laboratories, which produce rapid results but do not adhere to the strict requirements needed for accreditation at the ISO or WHO level.^1^ Biolab has a long‐standing commitment to delivering the highest quality laboratory testing and has attained accreditation in several areas, including enrolling in College of American Pathologists (CAP)/Clinical Laboratory Improvement Amendments programmes from the United States. This commitment was carried through to COVID‐19 testing, with three levels of validation required for each result to be confirmed as correct before reporting. The assay used contained an internal RNA control (MS2) which was included with each run to ensure that extraction was successful, while a PCR control, both positive and negative, was added post‐extraction to each plate to ensure PCR was optimal. The validation chain was then threefold: Primary assessment was done by the laboratory technician, who would check the PCR results, including the internal, positive and negative controls were correct. Secondary confirmation was done by the laboratory supervisor, who would confirm each plate was correct before releasing the results to the laboratory director. The laboratory director, who is an accredited medical specialist, would then review the results before release to the MoH and other stakeholders. This chain also included enrolling in the annual CAP External Quality Assurance programme where samples were provided in virus transport medium that where then extracted, amplified, and run with results reported to CAP in a timely fashion. Biolab attained “Good” grades (Table 1) in both assessments undertaken, with no CAP exceptions reported. This provides reassurance that the testing performed in these laboratories meet the highest possible standards like those done in other developed countries, including the United States.

**TABLE 1:** CAP External Quality Assurance programme results for COVID‐19 diagnostics, covering the peak of the pandemic between 2021 and 2022.

	Grade	CAP exceptions	Comment
2021	Good	Nil	All positive/negative correctly detected
2022	Good	Nil	All positive/negative correctly detected

*Note*: Biolab reported all samples correctly, with no exceptions reported. Further enrolment will be considered depending on the requirements for testing in Jordan through 2023 and beyond.

Abbreviation: CAP, College of American Pathologists.

### 3.3 Biolab played a key role in genomic surveillance in Jordan, providing >75% of all genomic samples submitted to GISAID in collaboration with international partners

**FIGURE 3:**
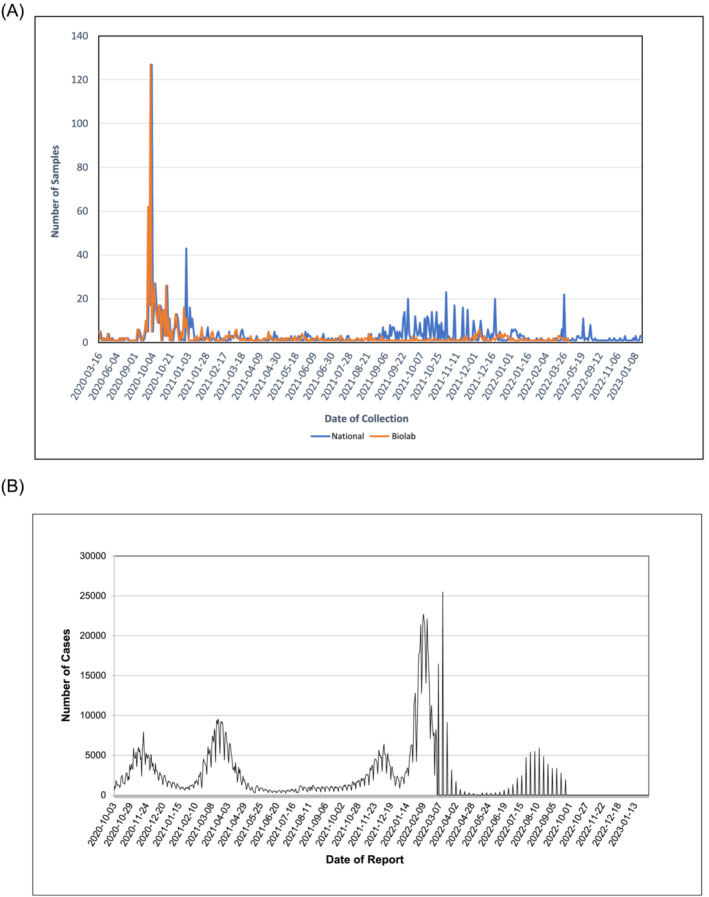
Biolab provided genomic surveillance support for the duration of the COVID‐19 pandemic. GISAID data were analysed to determine the frequency and number of samples submitted by Biolab and the country of Jordan as a whole from 2020 to 2023. (A) Samples are shown from the date of sample collection to allow direct comparison with daily testing results collated from the World Health Organization. In each case, Jordanian sequencing was operational prior to (B) global waves of infection, with the B.1.1.312 wave in 2020, alpha wave in early 2021, and delta wave in late 2021 before the omicron surge in 2022.

Biolab played a crucial role in Jordan, providing the earliest in‐country genomic sequencing reported in GISAID including the first reports of the vast majority of emerging Variants of Concern (VOC) (Figure 3). The laboratory collaborated closely with the Andersen laboratory at the Scripps Institute in California, both sending samples for sequencing and implementing in‐country workflows to build the capacity for Jordan to sequence viral samples as needed routinely. In the early stages of the pandemic, Biolab was the sole submitter to GISAID, with other laboratories contributing to the global effort routinely from late 2020 onwards. Overall, Biolab submitted 1086 sequences to GISAID, out of the total 1443 complete high coverage sequences in the database from Jordan (75.3% of total Jordanian samples in GISAID). These data have been analysed comprehensively elsewhere,^3^ clearly demonstrating the value that genomics provides during an outbreak or pandemic.

### 3.4 The measures implemented to support staff health and well‐being had an overall positive effect on staff engagement and attitudes

Laboratory professionals were one of the many dedicated workforces that continued to work throughout the COVID‐19 pandemic, supporting their medical and healthcare colleagues and the public by ensuring that they had access to sample testing and reporting rapidly. While this was commendable, with governments and the public providing some support, this clearly impacted laboratory staff's mental health and well‐being, with some countries reporting staff burnout and fatigue.^5^, ^6^ Biolab was aware of this issue and proactively provided staff with enhanced support and working conditions to ensure staff were protected and supported throughout the pandemic. Measures included salary, health, child support, and more, outlined in Section 2.

**FIGURE 4:**
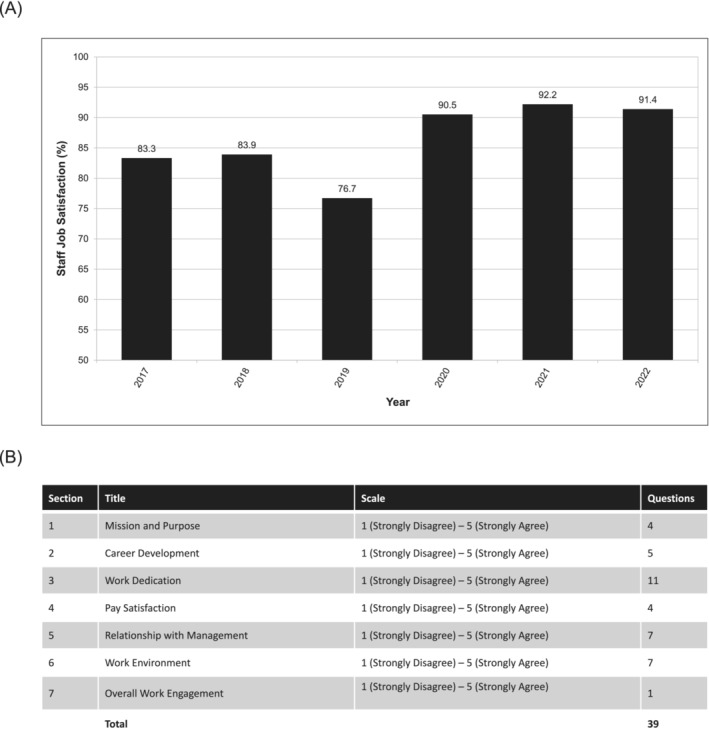
Staff satisfaction showed a year‐on‐year increase despite the COVID‐19 pandemic and increased workloads. (A) Overall, staff satisfaction remained high throughout the COVID‐19 pandemic in comparison with previous years ranging from 2016 to 2022. (B) Staff satisfaction was measured through a survey covering a range of topics, with a score given for each from 1 (*Strongly disagree*) to 5 (*Strongly agree*). Results were collected with the satisfaction calculated as the average score from each question (e.g., a score of 4 would be 80% satisfied, while 2 would be 40% satisfied, and then, the average of all 39 questions would be the “satisfaction” score).

To determine whether the measures were effective, Biolab HR monitored staff satisfaction through a series of surveys across the pandemic. The surveys gauged overall staff satisfaction with their place of employment (Figure 4). They sought feedback on the critical areas of improvement which would continue to maintain or improve their engagement with work (listed in Figure 4B). Overall, satisfaction increased yearly, peaking at 92.2% during 2021. Compared with the 76.7% satisfaction at the beginning of the pandemic, this represents a unique case of improved job satisfaction during the COVID‐19 pandemic, indicating that the measures taken by the Biolab team were effective.

Other than compensation, critical areas of satisfaction included the staff identifying that communication between the senior and junior staff was effective, that the staff felt engaged in their day‐to‐day work, and showed appreciation for scientific training provided beyond normal work. This investment by Biolab in staff well‐being forms an excellent model for staff retention and engagement and contributed to the high quality of the work done throughout the pandemic.

## 4 DISCUSSION

COVID‐19 dramatically impacted the world, placing burdens on health and laboratory infrastructure to keep up with the demand for testing to meet governmental requirements and recommendations. To maintain high‐quality, timely laboratory results, public and private entities had to find innovative solutions to problems such as logistics, a geographically diverse sampling and testing frequency, staff health, well‐being, and sustainability in the face of the vast amount of samples requiring testing. Jordan had a robust, well‐developed public health laboratory infrastructure, established over the years to respond to influenza, SARS‐CoV‐1, Middle East Respiratory Syndrome, and other pathogens of concern, but faced the same challenges as most countries, as the public health infrastructure was overwhelmed by the workload needed to keep up with travellers, test‐and‐trace systems, variant assessment, and other recommended actions that helped Jordan effectively monitor infection in the country.

Private and public laboratory collaborations are not new, with model systems in place like the United States, where the Centres for Disease Control and Prevention frequently rely on private laboratories to provide testing support across the country.^7^ However, Jordan does not have the resources or the diverse array of private testing facilities that countries such as the United States do nor do they have the integrated IT systems that are well established through the State and Federal authorities. The collaboration needed to be built from scratch, including establishing how to integrate sample testing and reporting through digital platforms which are not traditionally linked in Jordan.

Biolab was uniquely positioned to respond to this need, with a geographically diverse network of laboratories with experience in state‐of‐the‐art molecular diagnostics. The release of the genome for SARS‐CoV‐2 in December 2019 meant that research institutes could design and publish primers that allowed for detection of the virus,^8^ with the first primers published in late January 2020 followed by a rapid expansion in the availability of commercial PCR assays.^9^ Biolab's rigorous research and development programme made them well‐placed for automated assays and platforms that met the requirements for high throughput, reproducibility, and quality with minimal benchtop work. The investment in automation paid off, with an average turnaround time of 1.5 h from when a sample reached the laboratory, unmatched in other laboratory networks, regardless of the diverse locations in which the laboratories were operating.

Biolab was also able to implement variant surveillance using ThermoFisher TaqPath COVID‐19 and PrimalSeq‐Nextera XT platform,^3^ which allowed them to support the monitoring of variants of concern and variants under investigation. Variant surveillance is a key aspect of the COVID‐19 pandemic, given the intrinsic differences in pathogenicity and infectivity observed between the different emerging variants. The delta variant, for example, showed increased pathogenicity and infectivity compared with the alpha wave^10^ but was less transmissible than the subsequent omicron wave.^11^ Vaccine sensitivity and response were also monitored through variant assessment, with omicron, for example, being less sensitive to neutralization of transmission of the virus than the delta or alpha variants.^12^ However, the vaccine was highly effective at reducing the severity of disease against all waves of infection.^12^ Despite the stringent quarantine and control measures introduced to the country, Jordan experienced similar but delayed waves of infection to the rest of the world but was able to effectively monitor and respond due to the efforts of the laboratory and public health networks working in concert.

Genomic surveillance was another key aspect of Biolab's response to the pandemic. Whole genome sequencing has been one of the key tools in the fight against COVID‐19.^3^ It provides information on epidemiological links between cases and variant emergence, while also allows for monitoring of the effectiveness of diagnostic assays, such as the loss of sensitivity of assays targeting the S‐gene due to mutations of the virus, termed spike gene target failure.^13^ An unprecedented global effort has been underway to sequence SARS‐CoV‐2, with over 15 million genomes now deposited in the GISAID repository providing a resource for vaccine and therapeutic and diagnostic assay design. Recognizing early that Biolab had the technology but not the expertise to introduce whole genome sequencing, they proactively partnered with recognized global experts at the Scripps Institute, with Prof Kristian Andersen.^14^ Over ~12 weeks, the Biolab team received sufficient training in the wet laboratory and bioinformatics protocols needed for sequencing the virus on the Ion torrent platform and was able to contribute actively to the global genomics efforts.^3^ This expertise will now be expanded and sustained to provide genomics support for other pathogens with the goal of preventing outbreaks from spreading to epidemics or pandemics.

One of the key outputs of the collaboration between Biolab and the public health laboratory system was the implementation of a digital IT platform, which allows for bidirectional, near real‐time transfer of results and data between laboratories, healthcare facilities, public health authorities, and patients. The rapid transfer and collation of accurate data is a key aspect to providing the necessary information for public health actions, and a unified end‐to‐end approach such as that taken by Biolab means that all levels of healthcare, from the patient through to ministers, can access reports to monitor the situation nationally and see how this fits with the international situation.

Automation was preferred over manual solutions to limit the benchtop time and reduce the burden on staff during the pandemic, as Biolab placed a premium on staff health and well‐being with an understanding that the longevity of the workforce would be key during a sustained pandemic response. This was one of many interventions taken by Biolab, including incentivizing staff and providing extensive support to ensure staff could continue to perform while under considerable stress. Altogether, these investments paid off with an improvement in staff engagement and satisfaction to 91% compared with 76% before the pandemic. This model of support for staff has not been widely reported, with other healthcare systems, such as the National Health Services in the United Kingdom,^15^ showing the opposite trend and seeing remarkable decreases in staff retention and satisfaction at work due to the strenuous working conditions they faced. It is of importance that staff satisfaction and HRs are considered as integral parts of laboratory network implementation, as the success and sustainability of the network is driven largely by the staff and ensuring their needs are considered is a key aspect of the success of Biolab over the years.

The collaboration between the public and private sectors, in this case through Biolab, allowed for the operationalization of the national surveillance programme significantly faster than would have been possible if the laboratory network had to be expanded entirely through the public health system. The success of this collaboration was made possible through aligning methodologies used between public and private sectors (PCR kits and cyclers) and through linking private laboratory results and associated metadata with MoH servers in real time. While academic laboratories have the capacity to provide support, there is a time investment needed to ensure that the data provided are of clinical standard, rather than research‐based, as the working conditions between good laboratory practice are not required for research and can have an impact on the reliability of results. Private laboratories are well equipped and regulated to ensure that they meet the standards required for clinical reporting of data, which allowed patients to receive their results in a timely fashion and the government to take actionable decisions when the need arose.

Successful collaborative ventures always come with challenges and lessons learned. Some of the major challenges faced in the early phase of the pandemic included: no dedicated IT solutions to communicate with patients at high‐throughput level, manual procedures of low RNA extraction and PCR testing throughput, patient queues and delays in screening centres, low number of trained molecular biologists, and lack of experience in obtaining nasopharyngeal swabs. Such challenges were successfully handled 3–6 months down the pandemic with the introduction of many IT solutions, automated and quick high‐throughput extraction and PCR machines, time‐saving corona drive‐through testing, and with the training of tens of new employees on molecular techniques by experienced staff and hundreds on collecting proper nasopharyngeal swabs by experienced Ear, Nose, and Throat (ENT) doctors.

## 5 CONCLUSION

Biolab implemented a unique model not seen elsewhere in the Arab world during the COVID‐19 pandemic, whereby it upscaled and customized operations early on at various fronts and partnered effectively with the public sector, assuming an exemplary role in sharing national responsibility and informing governmental response.

Biolab managed to perform many PCR tests covering a wide geographic area and was an early adopter of next‐generation sequencing publishing more than three quarters of the total high coverage sequences submitted to GISAID specifically by Jordanian laboratories. This high scientific throughput would not have been possible without an equal development of IT and logistical solutions, quality assurance, and focus on staff well‐being by the HRs department.

Overall, the partnership between Biolab and the national public health and national academic bodies such as the MoH and the laboratory of the Princess Haya Center for Biotechnology, among others, has shown to be highly effective and forms an excellent prospective model for public and private health engagements during future outbreaks.

## AUTHOR CONTRIBUTIONS

I. A. D. conceived and designed the manuscript, contributed data, collected data, performed analysis, and wrote the paper. Z. N. contributed data, collected data, and performed analysis. L. M. designed the manuscript, performed analysis, and wrote the paper. L. A. wrote the paper. D. N. contributed data, collected data, and performed analysis. L. S. contributed data, collected data, and performed analysis. M. G. contributed data, collected data, and performed analysis. T. H. contributed data, collected data, and performed analysis. A. A. conceived and designed the manuscript, contributed data, performed analysis, and wrote the paper.

## CONFLICT OF INTEREST STATEMENT

The authors declare no conflict of interest.

### PEER REVIEW

The peer review history for this article is available at https://www.webofscience.com/api/gateway/wos/peer-review/10.1111/irv.13209.

## Data Availability

Data sharing is not applicable to this article as no new data were created or analysed in this study.
